# Sinbase 2.0: An Updated Database to Study Multi-Omics in *Sesamum indicum*

**DOI:** 10.3390/plants10020272

**Published:** 2021-01-30

**Authors:** Liwen Wang, Jingyin Yu, Yanxin Zhang, Jun You, Xiurong Zhang, Linhai Wang

**Affiliations:** 1 Key Laboratory of Biology and Genetic Improvement of Oil Crops, Oil Crops Research Institute of the Chinese Academy of Agricultural Sciences, Ministry of Agriculture, No.2 Xudong Second Road, Wuhan 430062, China; wangliwenjn@163.com (L.W.); jy526@cornell.edu (J.Y.); 13277065857@163.com (Y.Z.); youjunbio@163.com (J.Y.); zhangxr@oilcrops.cn (X.Z.); 2 Shandong Luyan Agricultural Co., LTC, Jinan 250100, China; 3 Boyce Thompson Institute, Ithaca, NY 14853, USA

**Keywords:** *Sesamum indicum*, genome, multi-omics, database

## Abstract

Sesame is one of the oldest oil crops in the world and is widely grown in the tropical and subtropical areas of Asia, Africa and America. Upon the completion of the sesame reference genome version 1.0, we launched Sinbase 1.0 as an integrated database for genomic and bioinformatics analyses. Recently, an upgraded version (version 2.0) of the genome sequence was released. In addition, large numbers of multi-omics data have been generated on sesame, but a comprehensive database that integrates these resources for the community has been lacking until now. Here, we developed an interactive and comprehensive sesame multi-omics database, Sinbase 2.0, which provides information of the sesame updated genome containing 13 chromosomes, 3 genetic linkage maps, 5 intra- and 6 inter-species comparative genomics, 1 genomic variation analysis, 5 transcriptome data, 1 proteome, 31 functional markers, 175 putative functional genes, and 54 QTLs detected for important agronomic traits. Moreover, Sinbase 2.0 has been enriched with novel user-friendly computational tools. All datasets of Sinbase 2.0 can be downloaded online conveniently. Sinbase 2.0 will be updated regularly with new available sesame multi-omics data and can be accessed freely via Sinbase 2.—Sesame Muti-Omics Database. We expect that Sinbase 2.0, similarly to the previous version, will continue to make a major contribution to advance sesame research towards a better understanding of its biology and genetic improvement, as well as comparative genomics and evolutionary biology.

## 1. Introduction

Sesame (*Sesamum indicum* L., 2n = 26) is one of the oldest oil crops in the world and is widely grown in the tropical and subtropical areas of Asia, Africa and America [1,2]. Sesame becomes a prized cash crop for small-scale farmers in developing countries because of its low production costs and high sale price. In addition, sesame is a very resilient crop able to provide yields and generate incomes on marginal lands and extreme climatic conditions. Therefore, sesame production experiences a strong growth and has attracted various studies from the scientific community on the genetic basis of important agronomic traits, essential for the improvement of the productivity and yield [3].

Over the past decade, the development of high-throughput sequencing technologies has promoted the study of sesame biology, generating invaluable multi-omics data. Foremost, the completion of the genome sequencing project of the modern cultivar “Zhongzhi13” (version 1.0) has ushered sesame research into a new era [4]. As sesame is gradually graduating from an “orphan crop” to a “genomic resource-rich crop”, several multi-omics data have been generated recently [3]. Different genome sequences of sesame landraces and modern cultivars have been released, providing opportunity for comparative genomics and pan-genome analysis [5,6,7]. Recently, the transcriptome data of sesame plants following drought stress and recovering periods was generated [8]. Another important abiotic stress was investigated by Wang et al. (2016) who reported large gene expression data under waterlogging stress in two contrasting sesame genotypes [9].

Several public databases including sesame genomics database (Sinbase 1.0) [10], sesame functional genomics database (SesameFG) [11] and sesame microsatellite marker database (SisatBase) [12], were then built to share sesame open data resources. However, SesameFG focused on some phenotypic and genotypic information, with SisatBase containing the information of microsatellite markers in sesame genome. So a comprehensive database that integrates the available multi-omics information for the community has still been lacking. Recently, the reference genome sequence was upgraded to reach 13 chromosomes (version 2.0), 94.3% of the estimated genome size and 97.2% of the predicted gene models [13]. Therefore, a new integrative and analytic web platform based on the version 2.0 of the sesame genome is critically needed. Here, we developed an interactive and comprehensive sesame multi-omics database, Sinbase 2.0 (http://www.sesame-bioinfo.org/Sinbase2.0), which provides information of the sesame updated genome (version 2.0), genetic linkage maps, comparative genomics, genomic variation, transcriptomics, proteomics, functional markers, genes, and QTLs. Moreover, Sinbase2.0 supplies user-friendly visualized and searchable tools, which can help users to retrieve sesame multi-omics information easily.

## 2. Implementation

**Figure 1:**
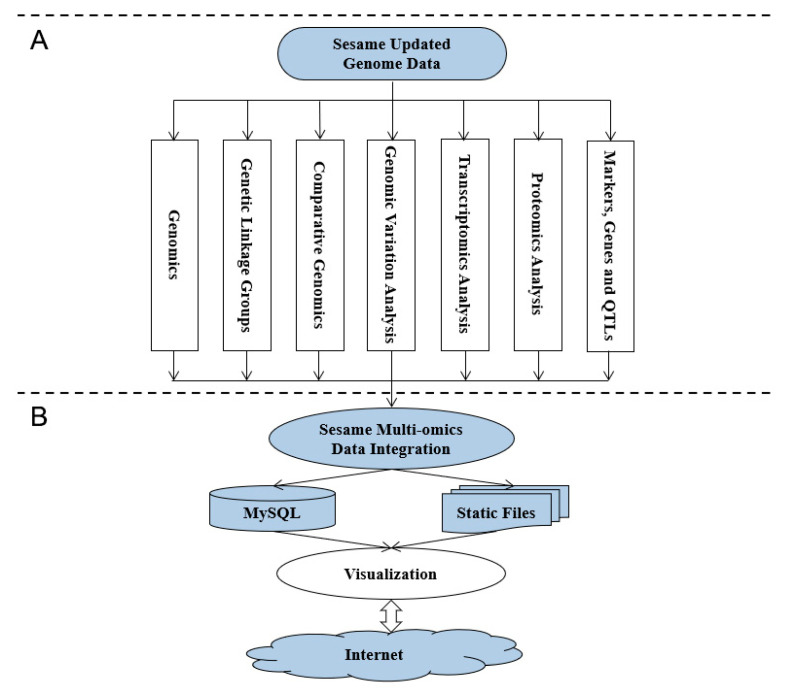
Framework for data integration and database implementation. (**A**) Data integration for sesame multi-omics data including the data of genomics; transcriptomics; proteomics; and functional markers, genes and QTLs. (**B**) Implementation of Sinbase 2.0 including data integration, data storage and visualization.

Sinbase 2.0 was implemented in a CentOS operation system with the Apache HTTP server and MySQL relational database management system. Web interfaces were developed by Perl, JavaScript and HTML programming languages. The graphical views of sesame chromosomes, interspecies comparative genomics, co-expressed genes in different transcriptomic experiments, and protein–protein interaction in proteomic experiment were drawn by Perl GD module from the Comprehensive Perl Archive Network (http://www.cpan.org/), Circos [14], WGCNA [15], and Cytoscape [16], respectively (Figure 1A). Basic datasets of sesame multi-omics were collected and analyzed by in-house Perl and Python scripts. These datasets were stored in the MySQL relational database, which can help users to retrieve useful information conveniently (Figure 1B).

## 3. Contents and Functions

In Sinbase 2.0, we integrated sesame updated genomics; genetics; comparative genomics; transcriptomics; proteomics; and functional markers, genes and QTLs using multiple-layer methods according to different multi-omics data formats.

### 3.1. Genomics

**Figure 2:**
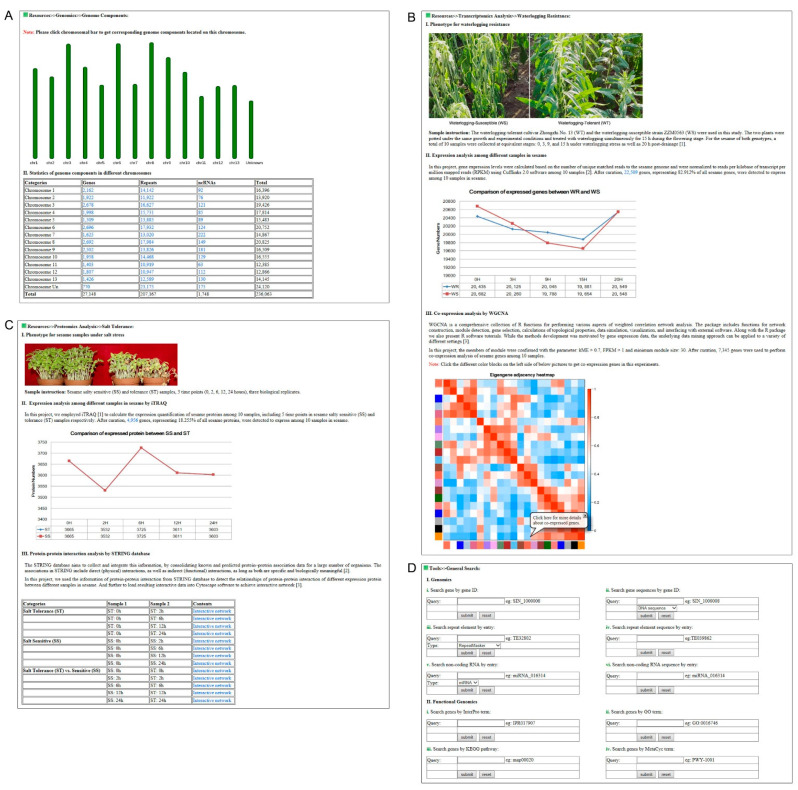
Screenshots of main functional pages in Sinbase 2.0. (**A**) Sesame genome components including protein-coding genes, repeat elements and non-coding RNAs. (**B**) Sesame transcriptomics data of waterlogging resistance including phenotype for waterlogging resistance, expression analysis among different samples in sesame and co-expression analysis by WGCNA. (**C**) Sesame proteomics data of salty tolerance including phenotype for sesame samples under salt stress, expression analysis among different samples in sesame by iTRAQ, and protein–protein interaction analysis by STRING database. (**D**) Keyword searching function including genome components and functional identifiers.

Concerning the genomic layer, we collected the sesame newly assembled genome version 2.0 and added newly annotated functionalities of putative protein-coding genes for genome-wide analysis [13]. We displayed genome components on sesame chromosomes including 27,148 protein-coding genes, 207,167 transposable elements and 1748 non-coding RNAs (Table 1). Each genome component was provided with detailed information containing basic description and sequence information (Figure 2A). All protein-coding genes were annotated by InterPro [17], Gene Ontology [18], KEGG [19], and MetaCyc [20] open-resource databases (Table 2). Moreover, for each protein-coding gene, the detailed information of transcriptomic and proteomic expression values, as well as homologous genes among different plant species based on comparative genomics analysis were supplied.

### 3.2. Genetics Linkage Analysis

For the genetic linkage maps, we supplied three types of molecular markers anchored on the sesame 13 chromosomes including 975 specific-locus amplified fragment sequencing (SLAF-seq), 347 simple sequence repeats (SSR) and 1522 bin markers. Each marker was given the genomic location information, two paired-ends primer sequences, product sizes, and upstream and downstream flanking genes.

### 3.3. Intra- and Inter-Species Comparative Genomics

Sinbase 2.0 also supplies intra-species and interspecies comparison for comparative genomic studies. Until now, there have been five released genomes of sesame including two landraces: Baizhima and Mishuozhima [6]; three modern cultivars: Zhongzhi13 [4], Yuzhi11 [7] and Swetha [5]. After curation of these genomes, we employed Burrows–Wheeler Aligner’s Smith–Waterman Alignment (BWA-SW) v0.7.15 to perform collinear analysis among the four genome-sequenced sesame varieties compared to the reference genome, which can help users to investigate the genomic variation within sesame varieties [21]. We used model plants and sesame close relative species to perform interspecies comparative genomics including Arabidopsis (Arabidopsis thaliana), rice (Oryza sativa), sorghum (Sorghum bicolor), tomato (Solanum lycopersicum), potato (Solanum tuberosum), and grape (Vitis vinifera). OrthoMCL v2.0.9 was employed to get orthologous gene groups among the seven plant species [22], and MCScanX was used to detect collinear genomic regions with collinear gene pairs between sesame and other plant species [23]. These analysis improved sesame gene functional annotations by mean of homologous relationships between the seven species.

### 3.4. Genomic Variation Analysis

Based on population genetic analysis of 705 sesame accessions, we obtained 12,833,863 single nucleotide polymorphism sites (SNPs) on the reference genome [6]. In Sinbase 2.0, SNPs can be searched by two ways including setting a range and a scale on sesame chromosomes. For each SNP, the location, reference and alternative sites, annotation, the gene to which it belongs to, and upstream/downstream gene list were supplied in detail.

### 3.5. Transcriptomics

For the transcriptomic module, we collected five transcriptome data released by our group including sesame waterlogging resistance [9], drought tolerance [8], three color seeds [4], determinate and indeterminate growth habit, and seed development transcriptomes. For each transcriptomic experiment, we supplied the phenotype information under different experimental conditions, expression analysis of sesame genes among different samples, as well as co-expression analysis of up- and down-regulated genes among different sesame samples employing WGCNA [15] (Figure 2B). Expression values of co-expressed genes among different samples and corresponding functions including COG [24], SWISS-PROT/TrEMBL [25], and nr annotations were further provided.

### 3.6. Proteomics

The proteomic module concerns proteomics data collected from sesame salt sensitive (SS) and tolerant (ST) cultivars during five time points (0, 2, 6, 12, 24 h) with three biological replicates based on iTRAQ analysis [26]. We supplied phenotype information of sesame SS and ST cultivars, expression analysis of proteins among different samples, and protein–protein interaction of up- and down-regulated proteins during the different time points (Figure 2C). Furthermore, users can retrieve the expression values of each sesame protein in different samples and protein–protein interactive networks of up- and down-regulated genes.

### 3.7. Functional Markers, Genes and QTLs

At last, 31 functional markers, 175 functional genes and 54 functional QTLs in the sesame genome, which control different agronomic traits including coloration, disease resistance, growth cycle, morphological characteristics, yield component, oil content and quality traits, and abiotic stress resistance, were gathered from the literature and mapped onto the sesame updated genome. We added the genomic location for each functional marker, two paired-ends primer sequences, the corresponding agronomic traits, as well as the upstream and downstream genes. We also joined hyperlink to the details of every functional gene. Next, we provided the location of functional QTLs and the gene list contained within each QTLs.

### 3.8. Useful Tools

To extract useful information and perform customized analyses on sesame multi-omics data, we developed and embedded several novel user-friendly tools in Sinbase 2.0 as compared with the previous version. General search was developed to get basic information of sesame genome components, gene functional annotation, homologous genes in relative species, and gene expression values in different experiments (Figure 2D). Customized BLAST was also embedded for users to get homologous genes or regions in the sesame reference and non-reference genomes by supplying protein or DNA sequence [27]. Localized MISAweb was included to identify SSR in the sesame reference and non-reference genomes [28]. Customized GBrowse [29] and GBrowse_syn [30] were embedded to display genomic components in sesame genome and collinear genomic regions between sesame and its close relatives, as well as model plants.

## 4. Utility

### Browse

Sinbase 2.0 supplied a multi-layer browsing function for users to extract useful information of sesame multi-omics. Users can browse sesame multi-omics information through “Resource” function of Sinbase 2.0, which includes genomics, genetics linkage analysis, intra- and inter-species comparative genomics, genomic variation analysis, transcriptomics analysis, proteomics analysis, markers, genes, and QTLs. Sesame different multi-omics information were collected in this section, which supplied a comprehensive annotation platform for functional exploration of sesame multi-omics data. For sesame genomics, users can get the latest sesame assembled genome sequences and function annotation with the latest versions of public open-resource functional databases, three types of genetic linkage groups based on sesame newly assembled genome sequences, intra- and inter-species comparative genomics, as well as genomic variation analysis among 705 sesame varieties from all over the world. For sesame transcriptomics, users can browse transcriptomic expression information of sesame protein-coding genes under different stress conditions, phenotypes, and growth and development stages. Moreover, users can get the co-expressed gene clusters and identical or interactive genes of interested sesame protein-coding genes focusing on same biological functions, key phenotypes or traits. For sesame proteomics, users can browse expression quantification of sesame proteins among sesame SS and ST samples. Moreover, users can get protein interactive information of interested sesame proteins under different treatments or different time periods.

## 5. Discussion

Sinbase 1.0 was the first practical and integrated database that focuses on the S. indicum genomics, genetics, and comparative genomics with its relatives and other important species in the plant community. It quickly became a reference genomic database for the scientific community working on sesame and related species. By providing the upgraded genomic sequences and more accurate genomic component annotation in the version 2.0 of Sinbase, we expect that users can perform functional genomics and comparative genomics studies in sesame with high precision and obtain more meaningful results. Compared with the Sinbase 1.0, Sinbase 2.0 supplies more comprehensive multi-omics datasets to allow users to study sesame thoroughly at the genetic and genomic levels. The genetic maps and molecular markers will be valuable resources for gene cloning and quantitative trait locus (QTL) detection. Transcriptome data and gene co-expression results will assist in genome-wide association studies and candidate gene mining. Since sesame entered the molecular breeding era, we believe that the functional genes, molecular markers and QTLs supplied in Sinbase 2.0 will assist breeders in the development of improved cultivars. Additionally, the user-friendly interface will allow users to access Sinbase 2.0 more conveniently. This database will be improved and updated continuously with new features, improvements to genome annotation and genomic sequences, and availability of more omics data.

## 6. Conclusions and Perspective

In summary, Sinbase 2.0 comprehensively integrates sesame multi-omics data including genomics, transcriptomics and proteomics; improves gene functional annotation; and provides a user-friendly functional analysis platform, which will bring more convenience for the scientific community. Functional modules of Sinbase 2.0 are useful for studying important issues of molecular biology, comparative and functional genomics, and molecular breeding in sesame. Sinbase 2.0 will be regularly updated with newly released sesame multi-omics data, and its gene functions will be refined with new versions of the sesame reference genome. As such, we hope that Sinbase 2.0, similarly to the previous version, will continue to make a major contribution to advance sesame research towards a better understanding of its biology and genetic improvement, as well as comparative genomics and evolutionary biology.

## Figures and Tables

**Table 1:** Statistics of genome components of Sesamum indicum genome v2.0.

Categories	Protein-Coding Genes	Repeat Elements	Non-Coding RNAs	Total
Chromosome 1	2162	14,142	92	16,396
Chromosome 2	1922	11,922	76	13,920
Chromosome 3	2678	16,627	121	19,426
Chromosome 4	1998	15,731	85	17,814
Chromosome 5	1509	13,885	89	15,483
Chromosome 6	2696	17,932	124	20,752
Chromosome 7	1625	13,020	222	14,867
Chromosome 8	2692	17,984	149	20,825
Chromosome 9	2502	13,826	181	16,509
Chromosome 10	1958	14,468	129	16,555
Chromosome 11	1403	10,919	63	12,385
Chromosome 12	1807	10,947	112	12,866
Chromosome 13	1426	12,589	130	14,145
Chromosome Un	770	23,175	175	24,120
Total	27,148	207,167	1748	236,063

**Table 2:** Statistics of functional annotation of protein-coding genes in Sesamum indicum genome v2.0.

Categories	Total Sesame Genes	Annotated Sesame Genes	Percentage (%)	Involved Terms or Identifiers
InterPro	27,148	20,009	73.70	5865
Gene Ontology		14,477	53.33	1780
KEGG		1985	7.31	122
MetaCyc		1351	4.98	577
